# Pupal and Adult Experience Affect Adult Response to Food Odour Components in the Flower-Visiting Butterfly *Tirumala limniace*

**DOI:** 10.3390/insects15040231

**Published:** 2024-03-27

**Authors:** Chengzhe Li, Hua Wang, Fangyuan Bian, Jun Yao, Lei Shi, Xiaoming Chen

**Affiliations:** 1 The Key Laboratory for Quality Improvement of Agricultural Products of Zhejiang Province, College of Advanced Agricultural Sciences, Zhejiang A & F University, Hangzhou 311300, China; chengzheli@163.com; 2 Department of Ecology, School of Life Sciences, Nanjing University, Nanjing 210023, China; wanghua@nju.edu.cn; 3 Key Laboratory of State Forestry and Grassland Administration on Bamboo Forest Ecology and Resource Utilization, China National Bamboo Research Center, Hangzhou 310012, China; bianfangyuan@yeah.net; 4 Institute of Highland Forest Science, Chinese Academy of Forestry, Kunming 650224, China; eyaojun@hotmail.com; 5 Research Center of Resource Insect, Chinese Academy of Forestry, Kunming 650224, China

**Keywords:** insect, behaviour, preimaginal experience, olfactory learning, antennal sensilla, electroantennogram, α-pinene, ethyl acetate

## Abstract

**Simple Summary:**

Butterfly larvae and adults can sense food odours from their surroundings, and exposure to these odours could alter larval or adult feeding preferences. However, it is still unknown whether butterfly pupae can sense odours from their surroundings. And little is known about how flower scents, in combination with food, affect butterfly adults’ subsequent foraging behaviour. *Tirumala limniace* (Lepidoptera: Danaidae) is a flower-visiting and highly charismatic butterfly. In this study, *T. limniace* pupae were exposed to the odour α-pinene and adults were exposed to the odours α-pinene and ethyl acetate to examine the effect of the experience on the foraging behaviour of *T. limniace*. The results showed that exposure of *T. limniace* pupae to α-pinene affected the feeding preference of newly emerged adults. *T. limniace* exhibits olfactory learning in the adult stage, and adult odour preferences are correlated with the frequency of their training, though an increased training time does not necessarily imply an enhanced learning ability. Unlike some studies in other systems, we found that *T. limniace* males learned odours faster than females. This may be due to differences in antennal sensilla, affecting sensitivity to odours and nectar demand between males and females. Our study can help elucidate the important role of learning behaviour in butterfly adaptive responses to the environment and lay the foundation for further research on butterfly learning and memory.

**Abstract:**

Butterflies have the ability to learn to associate olfactory information with abundant food sources during foraging. How the co-occurrence of both food and food odours affects the learning behaviour of adults and whether butterflies perceive the odour of their surroundings and develop a preference for that odour during the pupal stage have rarely been tested. We examined the effect of experience with food odour components (α-pinene and ethyl acetate) during the pupal and adult stages on the foraging behaviour of the flower-visiting butterfly *Tirumala limniace*. We found that α-pinene exposure during the pupal stage changed the foraging preference of newly emerged adults. *T. limniace* exhibits olfactory learning in the adult stage, and adult learning may influence their previous pupal memory. Moreover, adults’ odour preference did not continue to increase over multiple training times. The learning ability of adults for floral odours (α-pinene) was greater than that for non-floral odours (ethyl acetate). In contrast to previous studies, we found that males learned odours more efficiently than females did. This could be attributed to differences in antennal sensilla, affecting sensitivity to compounds and nectar demand between males and females. Our study provides further insight into how olfactory learning helps flower-visiting butterflies use food odours to forage better.

## 1. Introduction

All creatures live in a constantly changing environment. For insects living in such dynamic habitats, the ability to learn to associate visual or olfactory information with abundant food sources is crucial for survival and reproduction [1,2]. Learning is classified as a ‘modification of behaviour as a result of experience’, and it helps insects quickly acquire food, suitable host plants, and mates and even avoid predatory natural enemies during their short lifespans [2,3]. One kind of learning, experience-induced preference, can induce behavioural changes associated with feeding [4], oviposition [5,6,7], and mate choice [8] in herbivorous insects.

Butterflies use visual (flower colour, shape, and size) and olfactory cues (flower volatiles and nectar composition) to discover and choose nectar plants [9], but learning may alter these preferences. Butterflies have an innate colour preference, but when conditioned with food rewards, their colour preference for flowers can shift, i.e., through visual learning [10,11]. They also exhibit olfactory learning, the ability to associate floral scents with unconditioned stimuli (US, e.g., nectar) and to shift preferred floral scents accordingly, which facilitates their adaptive responses to complex and variable environments. Butterflies have different learning abilities in response to different odours. For example, in a study on sugar preference in *Agraulis vanilla*, both males and females were successfully conditioned when exposed to floral volatiles butyl acetate and amyl acetate but not to host plant (*Passiflora incarnata*) volatiles, suggesting that *A. vanilla* had a greater capacity to learn floral odours than host plant odours [12].

Olfactory learning occurs at different stages of insect ontogeny rather than only in the adult stage. Learned by experience, butterfly larvae feeding on host plants has been found to influence feeding preference in the larval stage [13] or to affect feeding preference [14] and host plant preference [7,15] in the adult stage. Thus, butterfly larvae exhibit olfactory learning, and this learning can influence adult behaviour after eclosion. However, not all butterflies learn equally well. A study on *Polygonia c-album* did not support the existence of olfactory learning in preimaginal butterflies [16]. There are many studies on butterflies with efficient olfactory learning in the larval and adult stages [3,12], but whether butterflies perceive the odour of their surroundings and develop a preference for that odour during the pupal stage has not been investigated.

Butterflies exhibit great diversity in sexual dimorphic learning abilities, and this diversity is one of the fascinating things about this large group of animals. For example, *A. vanilla* females are significantly more efficient at learning odours than males [12], while other studies have shown that both sexes of *Danaus plexippus* are equally good at learning odours [17]. However, whether there are butterfly species where males learn odours more efficiently than females is still unknown.

*Tirumala limniace* (the blue tiger, Lepidoptera: Danaidae), as a flower-visiting and highly charismatic butterfly, is utilized in butterfly gardens in China. The pupal stage of *T. limniace* lasts approximately 10 days, and the adult stage lasts approximately 15 days. Adults start to visit flowers on the second day after eclosion, and courtship and mating take place on the sixth day, while oviposition occurs the following day [18]. During foraging, *T. limniace* had some response to visual cues but gave more weight to olfactory cues [19].

In this study, we used *T*. *limniace* as the research object and mainly wanted to explore how experience affects adult foraging preferences. This study selected two chemical odours, α-pinene for floral volatiles and ethyl acetate for fruit volatiles, which were combined with sucrose solution for adult training. Pupae were exposed to the volatile α-pinene until they emerged. We wanted to determine (1) whether butterflies perceive and remember the odour of their surroundings during the pupal stage, (2) the effect of adult training time and frequency on adult learning ability, and (3) whether there is a difference between male and female adult learning ability for volatiles from the adults’ nectar and non-nectar plants. Answering these questions can help elucidate the important role of learning behaviour in butterfly adaptive responses to the environment and lay the foundation for further research on butterfly learning and memory.

## 2. Materials and Methods

### 2.1. Study Animals

*Tirumala limniace* individuals were obtained from artificial breeding populations in Yuanjiang County, Yunnan Province, China, and larvae were reared on the host plant *Dregea volubilis* under controlled conditions consisting of a temperature of 28 ± 2 °C, a 13L:11D photoperiod, and a relative humidity of 40–60%.

### 2.2. Compounds of Food Odours

The compounds of floral odours that attract *T. limniace* adults were screened based on the results of Tang [20]. Among the six tested compounds (α-pinene, 1-octanal, terpineol, methyl salicylate, benzaldehyde, and eucalyptol), α-pinene was found to be the most attractive and was chosen as the stimulus for learning and training during both the pupal and adult stages of *T. limniace*.

To investigate whether there are differences in the learning ability of male and female adults and differences in the learning ability of volatiles from their preferred and nonpreferred nectar plants, we selected α-pinene (preferred nectar plants) and ethyl acetate (EA, non-nectar plants, the main volatiles emitted from a wide range of fruits) [21] as adult learning training stimuli.

### 2.3. Pupal Odour Exposure

Two hundred pupae of the same size were selected after the larvae pupated. The experimental group consisted of 100 pupae placed on the partition of the desiccator (3 L), and 20 mL of 5% α-pinene aqueous suspension was added to the defatted cotton below the partition. The control group consisted of another 100 pupae placed in another desiccator (3 L), with 20 mL of distilled water added to the defatted cotton. The pupae did not come into contact with the defatted cotton. Both groups underwent exposure for 6 h each day until the day before eclosion. These pupae were exposed to α-pinene or solvent for 5 d. Then, the pupae were placed in a clean eclosion chamber and washed using distilled water and hexane to remove chemical cues on the surface of the pupae. Sixty newly emerged adults (♀:♂ = 1:1) were selected from each group and starved for 1 d before behavioural tests.

### 2.4. Adult Stage Experience

**Figure 1:**
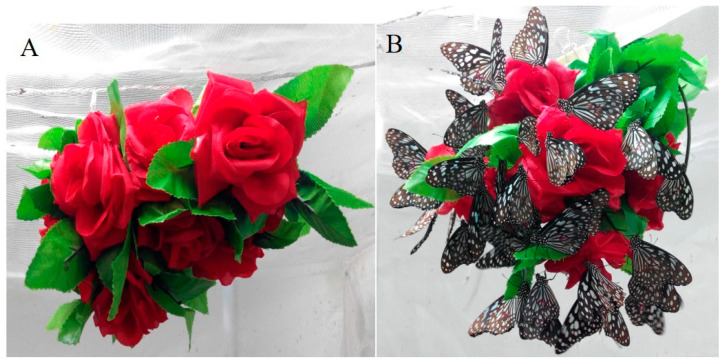
Red artificial flower clusters (**A**) and *Tirumala limniace* adults trained to feed on α-pinene- or solvent-containing flowers (**B**).

*T. limniace* adult learning was conducted in a net house (4 × 2 × 2 m) located in a temperature-controlled room (26 ± 2 °C, 13L:11D, 40–60% RH). To serve as feeding substrates, spherical artificial flower clusters (Figure 1A) were hung in the net house, and 0.5% α-pinene (or 0.5% ethyl acetate) + 15% sucrose solution was sprayed on the red flowers to feed the adults (Figure 1B). Adults feeding only on 15% sucrose solution were used as control groups. Each flower cluster was made up of a 2.5 m long green plastic stem with 10 red artificial flowers (silk cloth, diameter approximately 9 cm). Adults were trained for 1–5 days to explore their learning differences after training for different lengths of time, while they were trained for 2, 4, or 6 days to investigate the learning differences between males and females. The number of trained adults for each behavioural test was 60–80 (♀:♂≈1:1). Each day had three training sessions: 09:00, 13:00, and 18:00. During each training session, the adults were allowed to feed for 10 min, and those that did not feed were hand-fed by unrolling their proboscis with a dissecting pin. All adults were starved for 1 d before the behavioural tests.

### 2.5. The Effects of Adult Experience on Earlier Pupal Experience

*T. limniace* adults that underwent pupal stage learning (exposed to α-pinene or solvent during the pupal stage) were subjected to learning for 3 d (both groups feeding on red cloths with 0.5% α-pinene + 15% sucrose solution), as described above. Then, they were starved for 1 d before performing the behavioural tests, with 60 adults (♀:♂ = 1:1) in each group.

### 2.6. Adult Behavioural Assays

The adult behavioural tests were conducted in a netted cage (4 m × 2 m × 2 m) in a well-ventilated artificial climate chamber. Two equally sized spherical artificial flower clusters were hung on the top of the netted room, 2 m apart, for adult feeding choice. In the experimental group, each red artificial flower was sprayed with 2 mL of 5% stimuli + 15% sucrose solution, while in the control group, each red artificial flower was sprayed with 2 mL of 15% sucrose solution. Stimuli (α-pinene or ethyl acetate) were sprayed on red artificial flowers outside the climate chamber. Adult butterflies were marked with an oil-based marker, placed in a release cage, and released one by one at a distance of 2 m from the flower clusters. The number of visits the trained adults made to the artificial flower clusters was recorded for both the experimental and control groups. The adults were placed back in another release cage immediately after making their first choice. The positions of the flowering clusters in the experimental and control groups were switched after all the trained adults made their choices. Then, these adults were released again, and their choices were recorded. To investigate changes in the feeding behaviour of adults trained for 3 days from 0 to 60 min, adults were released and not retrieved, and they were allowed to feed for 60 min inside the net chamber. The number of visits made by the trained adults to each flower cluster was documented at 0, 5, 10, 20, 30, and 60 min following the release of the adults. To avoid the influence of odour residue, the netting near the flowering clusters in the experimental group was wiped with alcohol-soaked cotton balls before swapping the position of the flower clusters. One visit was defined as an adult landing on an artificial flower and extending the proboscis for feeding. The proportion of visits = (the number of adults visiting α-pinene-, EA- or solvent-containing flowers/the total number of released adults) × 100%. Adults that had not made a selection after 10 min were noted as ‘not selected’.

### 2.7. Scanning Electron Microscope

The antennae of 5 males and 5 females (2–3 d post-emergence) were removed under a microscope (Nikon SMZ1500, Tokyo, Japan) by using a blade. The antennae were washed for 30 s using 70% ethanol solution and stove-dried at 40 °C for 24 h. After drying, the specimens were attached to a holder using electric adhesive tape, sputter-coated with gold for 45 s (Hitachi e-1010, Tokyo, Japan), and examined and photographed with an S-3000 N SEM (at 16 kV, Hitachi, Tokyo, Japan). The different sensilla types were discriminated based on their morphological features described in the literature [22,23,24]. The abundance of different sensilla types of *T. limniace* varied, and, thus, we counted the sensilla on each segment from the dorsal, ventral, and lateral parts of the antenna.

### 2.8. Electroantennogram (EAG) Recordings

Antennae from 6 male and 6 female butterflies (2–3 d post-emergence, unmated) were used. The EAG techniques used in this study were similar to those described by Li et al. [25]. Glass capillaries (1.1 mm I.D.) filled with saline solution (4.7 mM KCl, 1.9 mM CaCl_2_, 130 mM NaCl) were used as electrodes. Antennal preparations were made by first cutting the base and distal ends of the antenna with a scalpel. The analogue signal was detected through a probe, captured and processed with a data acquisition controller (IDAC-2, Syntech, Kirchzarten, Germany), and later analysed using software (EAG 2014, Syntech, Hilversum, The Netherlands). Two dilutions (1 and 100 μg/μL) of two compounds (α-pinene and ethyl acetate, both purchased from Shanghai Aladdin Bio-Chem Technology Co., Ltd., Shanghai, China) were prepared in hexane. A 20 μL aliquot of each solution was applied to a piece of filter paper (10 × 30 mm). After allowing for solvent evaporation, the filter paper was inserted into a glass Pasteur pipette to form an odour cartridge. The control stimulus was a similar pipette containing filter paper treated with a 20 μL aliquot of hexane. The stimulation was delivered at a flow rate of 20 mL/s in a 0.5 s puff using a stimulation device (Syntech).

### 2.9. Statistical Analysis

The data were analysed using SPSS software version 18.0. The Kruskal–Wallis *H* test was used to examine significant differences in preference for artificial flowers after pupae and adults were exposed to α-pinene or solvent treatments and at different time points after the adults were released. The chi-square test was used to determine significant differences in the proportion of visiting flowers after adults were trained for 1–5 days. Student’s *t* test was used to determine significant differences in the EAG values between males and females at different concentrations of the same volatiles. The Mann-Whitney *U* test was used to determine significant differences in the total number of antennal sensilla between males and females. The proportion of individuals visiting artificial flowers was compared between males and females using a generalized linear model (GLM) with a binomial error distribution, with gender, training days, and their pairwise interactions as fixed factors and the proportion of visits as a continuous variable.

## 3. Results

### 3.1. Exposure to α-Pinene during the Pupal Stage Affects Adult Feeding Preference

**Figure 2:**
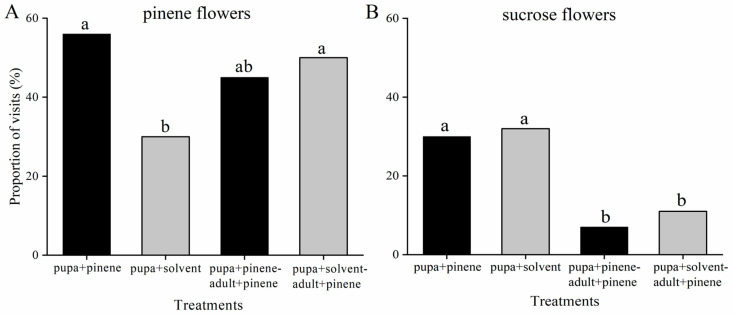
Effects of α-pinene exposure in the pupal stage on adult feeding preference. Percentage of adults visiting artificial flowers containing α-pinene + sucrose solution (**A**) or only sucrose solution (**B**). Pupa + pinene and pupa + solvent: the pupal stages were exposed to α-pinene or solvent, and the preference of the newly emerged adults was tested. Pupa + pinene-adult + pinene and pupa + solvent-adult + pinene: the pupal stages were exposed to α-pinene or solvent, respectively, and these adults were exposed to α-pinene for 3 d; then, their preferences were tested. Different letters indicate significant differences between treatments and controls (Kruskal–Wallis *H* test, *p* < 0.05).

*T. limniace* pupae were exposed to volatiles of α-pinene or solvent until they emerged. The percentage of newly emerged adults that had been exposed to the volatile α-pinene during the pupal stage and visited the α-pinene-containing flowers was significantly greater than that of the solvent-treated controls (χ^2^ = 4.35, df = 1, *p* = 0.04). However, there was no significant difference in the proportion of α-pinene- and solvent-treated adults that selected α-pinene-containing flowers after both groups were exposed to α-pinene for 3 days (χ^2^ = 0.26, df = 1, *p* = 0.61) (Figure 2A). There was no significant difference in the percentage of visits to solvent-containing flowers by newly emerged adults that had been exposed to α-pinene or solvent during the pupal stage (χ^2^ = 0.07, df = 1, *p* = 0.80). After these newly emerged adults were exposed to α-pinene for 3 days, there was still no significant difference in the proportion of adult visits to the solvent-containing flowers (χ^2^ = 0.90, df = 1, *p* = 0.35), while the proportion of visits was reduced compared to that of the newly emerged adults (Figure 2B). These results showed that α-pinene exposure during the pupal stage changed the preference of newly emerged adults, and that pupal odour exposure had little effect on the feeding preference of adults who experienced odour exposure.

### 3.2. Adult Learning Experience with α-Pinene Affects Feeding Preference

**Figure 3:**
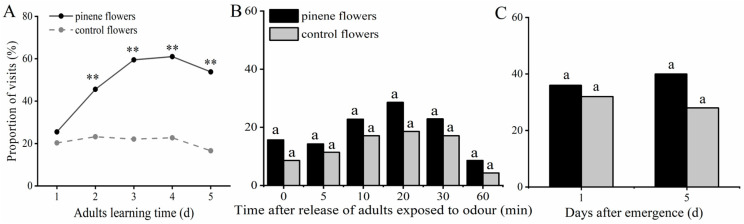
Effects of adult learning experience with α-pinene on feeding preference. (**A**) Percentage of adults visiting artificial flowers after training for 1–5 d; (**B**) percentage of adults (trained for 3 d) visiting artificial flowers during different periods after release; (**C**) percentage of naive adults (no learning experience) visiting artificial flowers on the 1st and 5th days after eclosion. Different letters indicate significant differences between treatments and controls (chi-square test, *p* < 0.05, ** *p* < 0.01).

After *T. limniace* adults experienced the floral volatile α-pinene + sucrose solution for 1–5 days, the percentage of adults visiting the α-pinene-containing flowers tended to first increase and then decrease with increasing training time, while the percentage of adults visiting the solvent-containing flowers remained unchanged at approximately 20% (Figure 3A). Except at the 1 d learning time point, the proportion of adult visits to α-pinene-containing flowers after 2–5 days of learning was significantly greater than that to solvent-only flowers (*p* < 0.01), indicating that *T. limniace* adults exhibit olfactory learning with the floral compound α-pinene.

After 3 days of training, *T. limniace* adults showed first increasing and then decreasing visitation rates to both flowers containing α-pinene + sucrose solution or sucrose solution from the beginning of release to 1 h. Visitation rates were high at the initial 10, 20, and 30 min and low at 0, 5, and 60 min (Figure 3B), indicating that flowers had different attraction effects on *T. limniace* adults at different times after these trained adults were released.

To test whether the preference of *T. limniace* adults for α-pinene is due to physiological maturation, behavioural experiments were carried out with adult butterflies that had no experience with α-pinene on the first and fifth days after emergence. There was no significant difference in the percentage of selection between α-pinene and control flowers by *T. limniace* adults on both the first and fifth days after emergence (1 d: χ^2^ = 0.24, df = 1, *p* = 0.63; 5 d: χ^2^ = 2.12, df = 1, *p* = 0.15) (Figure 3C). This shows that the changes in adult preferences for α-pinene are not due to physiological maturation but are instead the result of learning.

### 3.3. Odour Preferences from Exposure to α-Pinene or Ethyl Acetate (EA) in Males and Females

**Figure 4:**
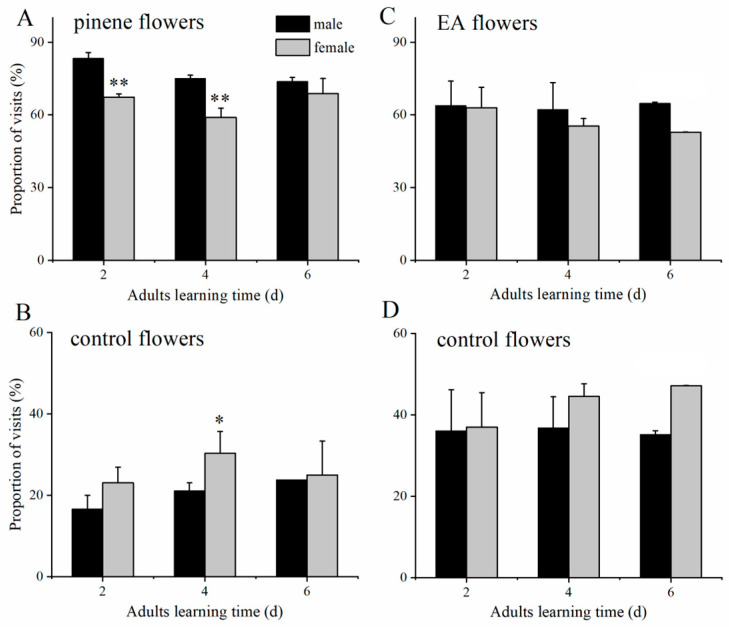
Effects of experience on the foraging preferences of *T. limniace* males and females after exposure to α-pinene (**A**,**B**) or ethyl acetate (EA; **C**,**D**) for a different duration (*n* = 4). Males and females were exposed to α-pinene or ethyl acetate for 2, 4, or 6 d; then, their preferences were tested. Generalized linear model (GLM), * *p* < 0.05; ** *p* < 0.01.

After 2, 4, and 6 days of exposure to α-pinene or EA, both males and females exhibited higher visitation rates to α-pinene- or EA-containing flowers than the control group (Figure 4). Significant main effects of sex (F_1,60_ = 30.03, df = 1, *p* < 0.001) and training days (F_2,60_ = 9.12, df = 2, *p* = 0.01) were evident; however, no significant interaction effect of sex × training days (F_2,60_ = 5.25, df = 2, *p* = 0.07) on the proportion of visits to α-pinene-containing flowers was detected. The visitation rate of males to α-pinene-containing flowers was significantly greater than that of females after 2 and 4 days of training (2 d: F_1,60_ = 16.76, df = 1, *p* < 0.001; 4 d: F_1,60_ = 16.86, df = 1, *p* < 0.001; Figure 4A). Conversely, females exhibited a stronger preference for sucrose flowers than males after a 4-day training period (F_1,60_ = 3.98, df = 1, *p* = 0.05; Figure 4B).

There was no significant main effect of sex (F_1,72_ = 2.91, df = 1, *p* = 0.09) or training days (F_2,72_ = 0.96, df = 2, *p* = 0.62) and no significant sex × training days interaction effect (F_2,72_ = 1.53, df = 2, *p* = 0.47) on the proportion of visits to EA-containing flowers. Following the 2-, 4-, and 6-day learning periods, there was no significant difference in the proportion of EA-containing flower visits between males and females (*p* > 0.05; Figure 4C,D), indicating that both *T. limniace* males and females are equally good at learning after exposed to EA. These findings indicate that *T. limniace* males learn α-pinene faster than females but not for EA.

### 3.4. Electrophysiological Responses of Males and Females to α-Pinene and Ethyl Acetate

EAG experiments were performed to investigate whether the feeding preference difference between males and females to the tested compounds α-pinene and ethyl acetate was related to the sensitivity of adults to the tested compounds. Our results showed that both α-pinene and ethyl acetate could induce EAG responses in male and female butterflies (Table 1). The EAG responses to low (1 μg/μL) and high concentrations (100 μg/μL) of α-pinene in males were significantly higher than those in females (1 μg/μL, t = 3.27, *p* = 0.01; 100 μg/μL, t = 2.63, *p* = 0.05), while there was no significant difference in EAG responses between males and females for both low and high concentrations of ethyl acetate (*p* > 0.05).

### 3.5. Morphology and Number of Antennal Sensilla in Males and Females

**Figure 5:**
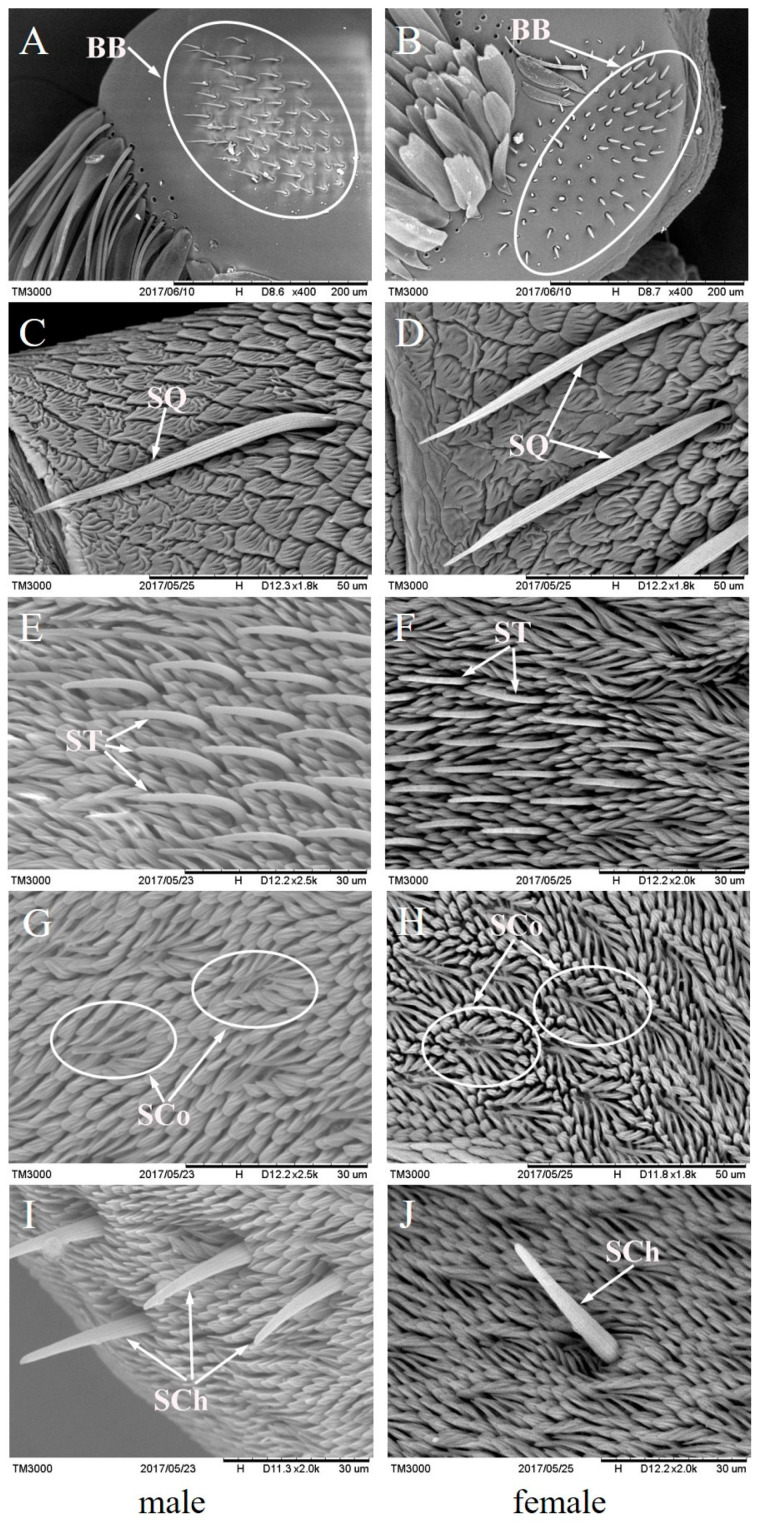
Antennal sensilla of *T. limniace*. (**A**,**B**) Böhm bristles (BB); (**C**,**D**) sensilla squamiformia (SQ); (**E**,**F**) sensilla trichodea (ST); (**G**,**H**) sensilla coeloconica (SCo); (**I**,**J**) sensilla chaetica (SCh).

The difference in the type and number of antennal sensilla in adults may also result in learning the difference between males and females. We found five types of sensilla on the antennae of both *T. limniace* males and females, i.e., Böhm bristles (BB), sensilla squamiformia (SQ), sensilla trichodea (ST), sensilla coeloconica (SCo), and sensilla chaetica (SCh) (Figure 5). There was no difference in the morphology of these antennal sensilla between males and females. However, the estimated numbers of ST and SCo in males were higher than those in females (ST: Z = −91.38, *p* < 0.001; SCo: Z = −220.84, *p* < 0.001), while the estimated number of SChs was higher in females than in males (Z = −29.13, *p* < 0.001) (Table 2).

## 4. Discussion

In this study, we found that, similar to the larval stage, exposure to food odours during the pupal stage of butterflies could alter the feeding preference of the newly emerged adults. We report for the first time that butterfly pupae can sense olfactory information present in their surroundings, and the newly emerged adults can maintain the olfactory information in memory of the pupal stage. *T. limniace* exhibits olfactory learning in the adult stage, and adults’ odour preferences are correlated with the frequency of their training, though an increased training time does not necessarily imply an enhanced learning ability. Both *D. plexippus* and *Byasa alcinous* males exhibit inferior visual learning [10,26], and *A. vanillae* males show inferior olfactory learning compared to females [12], while both sexes of *D. plexippus* are equally good at learning odours [17]. However, our study found that the olfactory learning of *T. limniace* is more prominent in males than in females. These differences are interesting and important to ecological diversity and evolutionary history across butterflies.

We observed no significant difference in the selection percentage between α-pinene and control flowers by adults with no prior exposure to α-pinene, on both the first and fifth days post-emergence (Figure 3C). However, after training for 2–5 days, the adults exhibited a preference for visiting α-pinene-containing flowers over control flowers (Figure 3A), which indicates the existence of olfactory learning in *T. limniace* adults. Interestingly, *T. limniace* adults had the highest visitation rates with the α-pinene + sucrose solution on days 3~4 of training, while the visitation rates decreased after 5 days (Figure 3A). This may be due to the fact that after 5 days of training, adults are in their courtship and mating stage [18], when courtship and copulation are their primary tasks and feeding is secondary. Adults at this stage may be more sensitive to sex pheromones and less sensitive to floral volatiles associated with foraging. Following a 3-day training period, the visitation rates of adults to artificial flowers were low at 0, 5, and 60 min (Figure 3B). This could be attributed to the released adults needed a few minutes to adapt to the new environment; for example, 60 min later, these adults may have been satiated and no longer needed to feed, or the stimuli (including food) may have no longer existed, resulting in lower visitation rates. Therefore, the odour preference of adults was correlated with training times, and the odour preference did not necessarily increase with an increase in training times.

Since the proportion of visits to α-pinene-containing flowers was higher than to EA-containing flowers in both males and females after exposure to odours for a different duration (Figure 4A,C), we suggest that *T. limniace* adults learned better with the floral volatile α-pinene than the non-floral volatile ethyl acetate. Kroutov et al. [12] also found that male and female *A. vanillae* were able to be conditioned to chemical stimuli of floral volatiles (amyl acetate and butyl acetate) but not of host plant volatiles. Li et al. [18] found differences in *Helicoverpa armigera* adult associative learning with two key floral odours; adults are more likely to associate phenylacetaldehyde and less likely to associate phenylmethyl acetate with sucrose solutions. These studies showed the importance of choosing the right floral volatiles for adult olfactory learning to achieve a better learning effect.

Several studies have found that female butterflies learn colour [26,27] and odour [12] more efficiently than males. In contrast to those results, we found that *T. limniace* males learned odours more efficiently than females. The main proximate reason may be the sensitivity of males and females to the tested compounds. We found that the EAG responses to low and high concentrations of α-pinene in males were higher than those in females (Table 1), indicating that males are inherently more sensitive to some odour compounds than females. In addition, the perception of food odours by adult butterflies is thought to be mediated by a combination of sensilla squamiformia, sensilla trichodea, and sensilla coeloconica on the antennae [23,24]. In this study, we found that the number of sensilla trichodea and sensilla coeloconica in the antennae of *T. limniace* males was higher than that of females; thus, the number of antennal sensilla in males and females may be associated with their learning differences. The ultimate reason may be the differences in nectar demands of male and female adults. The major life activities of male butterflies revolve around courtship and mating. During the first to sixth day after eclosion, *T. limniace* males focus on flight and flower visits [18], which promote reproductive organ development through flight [28] and nutritional supplementation [29]. Moreover, male butterflies rely on flight to practice courtship and find their mates, and flight requires a supply of energy. Li et al. [18] studied the flower-visiting behaviour of *T. limniace* adults and found that males visited more flowers than females on days 1–6 after eclosion, both in terms of the average foraging frequency and foraging duration; that is, the nectar demand of males exceeded that of females on days 1–6 after eclosion.

The phenomenon of memory persisting through metamorphosis, i.e., preimaginal memory persists into the adult form, has been reported in several insects, such as flies [30,31], parasitic wasps [32], moths [33], and ants [34]. Gowri and Monteiro [35] found that *B. anynana* larvae learned to prefer the novel odour within a generation of odour feeding and transmitted the learned preference to the next generation. We found the newly emerged *T. limniace* adults could maintain the memory experienced during the pupal stage. These results indicated that this phenomenon also exists in butterflies. Mushroom bodies are central to olfactory learning and memory in butterflies and other insects [34,36]. Very little is known about the structural modification of butterflies’ brains during development and about pupal olfactory systems. Though the underlying processes are largely unknown, mushroom body cells’ survival through metamorphosis has been indicated as a possible mechanism [30].

In conclusion, we demonstrated that odour exposure of *T. limniace* in both the pupal and adult stages could influence the subsequent foraging preference of adults. An increased duration and frequency of adult training is not necessarily associated with increased odour preferences. However, experiences during the adult stage may mask or influence their previous experiences in the pupal stage. Similar studies, such as Stanton’s [37], also found that butterflies’ nectar foraging in some way interferes with the retention or retrieval of stored information about host plants (an earlier experience), indicating that a novel learning experience appears to interfere with recall of an earlier experience. Perhaps butterflies are somehow neurally restricted in their ability to store or use such information [38]. *T. limniace* males learned food odours more efficiently than females, which may be caused by the differences in the sensitivity to odours, in antennal sensilla, and in nectar demand between males and females.

## Figures and Tables

**Table 1:** Electroantennogram (EAG) responses (mV, mean ± SE; *n* = 6) of *T. limniace* females and males to low and high doses of two compounds.

Compounds	1 μg/μL		100 μg/μL	
	♀	♂	♀	♂
α-pinene	0.98 ± 0.13Ab	1.92 ± 0.25Aa	0.95 ± 0.16Ab	2.09 ± 0.41Aa
ethyl acetate	1.25 ± 0.23Aa	1.99 ± 0.32Aa	1.36 ± 0.17Aa	2.04 ± 0.35Aa

Capital letters next to the data show the results of lengthwise comparisons, and small letters show the results of crosswise comparisons. The same letter indicates no significant difference (Student’s *t* test, *p* > 0.05), and different letters indicate significant differences (Student’s *t* test, *p* < 0.05).

**Table 2:** Estimated number of sensilla (mean ± SE; *n* = 5) in the antennae of *T. limniace* males and females.

	Böhm Bristles (BB)	Sensilla Squamiformia (SQ)	Sensilla Trichodea (ST)	Sensilla Coeloconica (SCo)	Sensilla Chaetica (SCh)
♂	146.0 ± 25.5A	27.5 ± 0.7A	2924.5 ± 287.8A	16,583.0 ± 222.0A	251.5 ± 12.0B
♀	153.0 ± 25.5A	33.0 ± 2.8A	2343.0 ± 11.3B	14,047.5 ± 185.5B	280.5 ± 20.5A

Capital letters show the results of lengthwise comparisons. The same letter indicates no significant difference (Mann-Whitney *U* test, *p* > 0.05), and different letters indicate significant differences (Mann-Whitney *U* test, *p* < 0.05).

## Data Availability

The data that support the findings of this study are available on figshare: https://figshare.com/s/0ecd15c3e440bd5b8aa5 (accessed on 14 February 2024).

## References

[1] Fan R.J., Anderson P., Hansson B.S. (1997). Behavioural analysis of olfactory conditioning in the moth *Spodoptera littoralis* (Boisd.) (Lepidoptera: Noctuidae). J. Exp. Biol..

[2] Jones P.L., Agrawal A.A. (2017). Learning in insect pollinators and herbivores. Annu. Rev. Entomol..

[3] Anderson P., Anton S. (2014). Experience-based modulation of behavioural responses to plant volatiles and other sensory cues in insect herbivores. Plant Cell Environ..

[4] Zhang H., Shan S., Gu S., Huang X., Li Z., Khashaveh A., Zhang Y. (2020). Prior experience with food reward influences the behavioral responses of the honeybee *Apis mellifera* and the bumblebee *Bombus lantschouensis* to tomato floral scent. Insects.

[5] Liu S.S., Li Y.H., Liu Y.q., Zalucki M.P. (2005). Experience-induced preference for oviposition repellents derived from a non-host plant by a specialist herbivore. Ecol. Lett..

[6] Kemp D.J. (2019). Manipulation of natal host modifies adult reproductive behaviour in the butterfly *Heliconius charithonia*. P. Roy. Soc. B-Biol. Sci..

[7] Braem S., Van Dyck H. (2023). Larval and adult experience and ecotype affect oviposition behavior in a niche-expanding butterfly. Behav. Ecol..

[8] Dion E., Pui L.X., Weber K., Monteiro A. (2020). Early-exposure to new sex pheromone blends alters mate preference in female butterflies and in their offspring. Nat. Commun..

[9] Barragán-Fonseca K.Y., Van Loon J.J., Dicke M., Lucas-Barbosa D. (2020). Use of visual and olfactory cues of flowers of two brassicaceous species by insect pollinators. Ecol. Entomol..

[10] Blackiston D., Briscoe A.D., Weiss M.R. (2011). Color vision and learning in the monarch butterfly, *Danaus plexippus* (Nymphalidae). J. Exp. Biol..

[11] Balamurali G., Rose S., Somanathan H., Kodandaramaiah U. (2020). Complex multi-modal sensory integration and context specificity in colour preferences of a pierid butterfly. J. Exp. Biol..

[12] Kroutov V., Mayer M., Emmel T. (1999). Olfactory conditioning of the butterfly *Agraulis vanillae* (L.) (Lepidoptera, Nymphalidae) to floral but not host-plant odors. J. Insect Behav..

[13] Silva A.K., Gonçalves G.L., Moreira G.R.P. (2014). Larval feeding choices in heliconians: Induced preferences are not constrained by performance and host plant phylogeny. Anim. Behav..

[14] Bernays E., Weiss M. (1996). Induced food preferences in caterpillars: The need to identify mechanisms. Entomol. Exp. Appl..

[15] Gowri V., Dion E., Viswanath A., Piel F.M., Monteiro A. (2019). Transgenerational inheritance of learned preferences for novel host plant odors in *Bicyclus anynana* butterflies. Evolution.

[16] Janz N., Söderlind L., Nylin S. (2009). No effect of larval experience on adult host preferences in *Polygonia c-album* (Lepidoptera: Nymphalidae): On the persistence of Hopkins’ host selection principle. Ecol. Entomol..

[17] Gegear R.J. (2021). Exploring the role of cognition in the annual fall migration of the monarch butterfly (*Danaus plexippus*). Insects.

[18] Li C., Wang F., Chen X., Zhou C., Yao J. (2015). Adult behavior of *Tirumala limniace* (Lepidoptera: Danaidae). J. Insect Sci..

[19] Tang Y., Zhou C., Chen X., Zheng H. (2013). Visual and olfactory responses of seven butterfly species during foraging. J. Insect Behav..

[20] Tang Y. (2013). The Research of Olfactory Andvisualresponses·during Butterflies Foraging.

[21] Lin M., Chen J., Wu D., Chen K. (2021). Volatile profile and biosynthesis of post-harvest apples are affected by the mechanical damage. J. Agric. Food Chem..

[22] Schneider D. (1964). Insect antennae. Annu. Rev. Entomol..

[23] Yuan X., Gao K., Yuan F., Zhang Y. (2014). Ultrastructure of antennal sensilla of four skipper butterflies in *Parnara* sp. and *Pelopidas* sp. (Lepidoptera, Hesperiidae). ZooKeys.

[24] Limberger G.M., Brugnera R., da Fonseca D.B. (2021). Antennal morphology and sensilla ultrastructure of *Ascia monuste* (Linnaeus) (Lepidoptera: Pieridae). Micron.

[25] Li C., Wang H., Chen X., Yao J., Deng J. (2022). Visual cues and body volatile β-ocimene are used by the blue tiger butterfly *Tirumala limniace* to identify conspecifics during courtship. Behav. Ecol. Sociobiol..

[26] Kandori I., Yamaki T. (2012). Reward and non-reward learning of flower colours in the butterfly *Byasa alcinous* (Lepidoptera: Papilionidae). Naturwissenschaften.

[27] Kandori I., Yamaki T., Okuyama S.-i., Sakamoto N., Yokoi T. (2009). Interspecific and intersexual learning rate differences in four butterfly species. J. Exp. Biol..

[28] Rankin M., Burchsted J. (1992). The cost of migration in insects. Annu. Rev. Entomol..

[29] Karlsson B., Wickman P.-O. (1990). Increase in reproductive effort as explained by body size and resource allocation in the speckled wood butterfly, *Pararge aegeria* (L.). Funct. Ecol..

[30] Ray S. (1999). Survival of olfactory memory through metamorphosis in the fly *Musca domestica*. Neurosci. Lett..

[31] Truman J.W., Price J., Miyares R.L., Lee T. (2023). Metamorphosis of memory circuits in *Drosophila* reveals a strategy for evolving a larval brain. Elife.

[32] Gandolfi M., Mattiacci L., Dorn S. (2003). Preimaginal learning determines adult response to chemical stimuli in a parasitic wasp. Proc. R. Soc. Lond. B Biol. Sci..

[33] Blackiston D.J., Silva Casey E., Weiss M.R. (2008). Retention of memory through metamorphosis: Can a moth remember what it learned as a caterpillar?. PLoS ONE.

[34] Signorotti L., Jaisson P., d’Ettorre P. (2014). Larval memory affects adult nest-mate recognition in the ant *Aphaenogaster senilis*. P. Roy. Soc. B-Biol. Sci..

[35] Gowri V., Monteiro A. (2024). Acquired preferences for a novel food odor do not become stronger or stable after multiple generations of odor feeding in *Bicyclus anynana* butterfly larvae. Ann. N. Y. Acad. Sci..

[36] Couto A., Young F.J., Atzeni D., Marty S., Melo-Flórez L., Hebberecht L., Monllor M., Neal C., Cicconardi F., McMillan W.O. (2023). Rapid expansion and visual specialisation of learning and memory centres in the brains of Heliconiini butterflies. Nat. Commun..

[37] Stanton M.L. (1984). Short-term learning and the searching accuracy of egg-laying butterflies. Anim. Behav..

[38] Papaj D.R., Prokopy R.J. (1989). Ecological and evolutionary aspects of learning in phytophagous insects. Annu. Rev. Entomol..

